# Title: Multi-Omics and Immune Landscape of Proliferative LncRNA Signatures: Implications for Risk Stratification and Immunotherapy in Hepatocellular Carcinoma

**DOI:** 10.3389/fphar.2022.907433

**Published:** 2022-05-18

**Authors:** Chi Liu, Jie Gao, Dongjing Yang, Qiwen Yu, Shuijun Zhang

**Affiliations:** ^1^ Department of Hepatobiliary and Pancreatic Surgery, The First Affiliated Hospital of Zhengzhou University, Zhengzhou, China; ^2^ Henan Engineering & Research Center for Diagnosis and Treatment of Hepatobiliary and Pancreatic Surgical Diseases, Zhengzhou, China; ^3^ Henan Research & Development International Joint Laboratory for Organ Transplantation Immunomodulation, Zhengzhou, China; ^4^ Zhengzhou Engineering Laboratory for Organ Transplantation Technique and Application, Zhengzhou, China; ^5^ Zhengzhou Key Laboratory for Hepatobiliary and Pancreatic Diseases and Organ Transplantation, Zhengzhou, China

**Keywords:** hepatocellular carcinoma, proliferation, multi-omics, machine learning, lncRNA

## Abstract

**Background:** Long noncoding RNAs (lncRNAs) are significantly implicated in tumor proliferation. Nevertheless, proliferation-derived lncRNAs and their latent clinical significance remain largely unrevealed in hepatocellular carcinoma (HCC).

**Methods:** This research enrolled 658 HCC patients from five independent cohorts. We retrieved 50 Hallmark gene sets from the MSigDB portal. Consensus clustering was applied to identify heterogeneous proliferative subtypes, and the nearest template prediction (NTP) was utilized to validate the subtypes. We introduced an integrative framework (termed “ProLnc”) to identify proliferation-derived lncRNAs. Moreover, a proliferation-related signature was developed and verified in four independent cohorts.

**Results:** In 50 Hallmarks, seven proliferation pathways were significantly upregulated and correlated with a worse prognosis. Subsequently, we deciphered two heterogeneous proliferative subtypes in TCGA-LIHC. Subtype 2 displayed enhanced proliferative activities and a worse prognosis, whereas subtype 1 was associated with hyperproliferative HCC and a favorable prognosis. The NTP further verified the robustness and reproducibility of two subtypes in four cohorts derived from different platforms. Combining the differentially expressed lncRNAs from two subtypes with proliferative lncRNA modulators from our ProLnc pipeline, we determined 230 proliferation-associated lncRNAs. Based on the bootstrapping channel and the verification of multiple cohorts, we further identified ten lncRNAs that stably correlated with prognosis. Subsequently, we developed and validated a proliferative lncRNA signature (ProLncS) that could independently and accurately assess the overall survival (OS) and relapse-free survival (RFS) of HCC patients in the four cohorts. Patients with high ProLncS score displayed significantly genomic alterations (e.g., TP53 mutation, 8p23-8p24 copy number variation) and higher abundances of immune cells and immune checkpoint molecules, which suggested immunotherapy was more suitable for patients with high ProLncS score.

**Conclusion:** Our work provided new insights into the heterogeneity of tumor proliferation, and ProLncS could be a prospective tool for tailoring the clinical decision and management of HCC.

## Introduction

Global Cancer Statistics 2020 demonstrated that hepatocellular carcinoma (HCC) ranks sixth in incidence and second in mortality (Sung et al., 2021). Although there are many treatment options for HCC patients, such as radical surgery, transcatheter arterial chemoembolization (TACE) therapy, targeted therapy, chemotherapy, and immunotherapy, the recurrence and mortality rates remain unsatisfactory (Craig et al., 2020; Cheng, 2021). The HCC management mainly relies on the clinicopathological stage. Existing studies have found significant heterogeneity in the risk of recurrence and death, even among patients at the same stage (Bou-Nader et al., 2020; Beaufrere et al., 2021). This is mainly because these classification systems only consider the clinicopathological factors but ignore the tumor’s molecular characteristics, and this inadequacy might be a potential cause of overtreatment or undertreatment in the clinic (Qian et al., 2021). Hence, developing a novel tool to improve the clinical outcomes of HCC patients is necessary.

The enormous development in high-throughput sequencing techniques has brought the molecular study of tumors into a new era and facilitated the breaking of genomic codes over the last few decades (Aizarani et al., 2019; Calderaro et al., 2019). These technologies provided new perspectives to explore the heterogeneity of tumors (Chia and Tan, 2016). Previous scholars have revealed the heterogeneity of HCC in terms of immune microenvironment, hypoxia, ferroptosis, and metabolism by analyzing the expression profiles (Huo et al., 2021). The intra- and inter-tumor heterogeneity of HCC might be the primary driver of distinct clinical outcomes in patients with the same stage. As previously reported, the different clinical outcomes might be mirrored at the heterogeneous molecular and cellular level (Liu et al., 2021a). Koncina et al. thought biomarkers were only considered appropriate if their expression was the same between and within tumor tissues, as they will then be expressed robustly in all patients. Therefore, a multigene panel might be a promising tool to address both inter- and intra-tumor heterogeneity (Koncina et al., 2020).

Recently, a novel class of non-coding RNA (lncRNAs) has been significantly implicated in tumorigenesis, progression, microenvironment, relapse, and prognosis of tumors (Klingenberg et al., 2017; DiStefano and Gerhard, 2021). For example, ID2-AS1 could suppress tumor metastasis *via* advancing the HDAC8/ID2 pathway (Zhou et al., 2020). ZFPM2-AS1 promotes HCC progression by activating the miR-653/GOLM axis and predicts worse prognosis (Zhang et al., 2021). DUBR induced by SP1 could facilitate oxaliplatin resistance of HCC by E2F1-CIP2A feedback (Liu S. et al., 2021). However, in the era of big data, proliferation-derived lncRNAs and their latent clinical significance remain largely unrevealed in hepatocellular carcinoma (HCC).

In our research, based on 50 Hallmarks, we identified seven proliferation pathways that upregulated and correlated with worse prognosis in HCC. Subsequently, we deciphered and verified two heterogeneous proliferative subtypes. The two subtypes displayed significant differences in proliferation state, prognosis, and underlying biological processes. A novel pipeline (termed “ProLnc”) was further introduced to recognize the latent lncRNA modulators of proliferation pathways. Ultimately, a proliferative lncRNA signature that possessed robust and accurate performance in assessing the prognosis of HCC was constructed and validated in our study.

## Methods and Materials

### Data Generation and Processing

The RNA-seq raw count data of HCC and adjacent normal tissues were obtained from The Cancer Genome Atlas database (TCGA-LIHC). Three microarrays, including GSE76427, GSE116174, and GSE144269, were generated from Gene Expression Omnibus (GEO) database. Additionally, the E-TABM-36 dataset was acquired from the ArrayExpress database. Patients with primary HCC, complete survival information and expression profile were enrolled in this study. The *aff*y and *Lumi* packages were applied to normalize microarrays from Affymetrix and Illumina companies, respectively. Collectively, the TCGA-LIHC dataset included 50 adjacent normal tissues and 351 HCC tissues, GSE76427, GSE116174, GSE144269, and E-TABM-36 datasets included 115, 64, 68, and 60 eligible HCC tissues, respectively.

### Hallmark Gene Sets

The Molecular Signature Database contains 50 Hallmark gene sets retrieved by previous researchers (Liberzon et al., 2015). The Hallmark gene sets were broadly applied in tumor-associated researches (Liu et al., 2021c). The gene set variation analysis (GSVA) was used to quantify the pathway activities.

### Consensus Clustering

Consensus clustering was an unsupervised machine learning algorithm, which provided a quantitative indication for determining the number and membership of potential clusters within a dataset. This approach was executed in the *ConsensusClusterPlus* package. The possible clustering number was set to 2-9, and this procedure relies on the K-mean clustering method and Euclidean distance was iterated 1000 times. The valid evidence was provided from distinct perspectives, including cumulative distribution function (CDF), the consensus matrix, and the proportion of ambiguous clustering (PAC). The silhouette statistic was employed to measure the affinity of samples in the cluster (Lovmar et al., 2005), which was a graphical aid to the interpretation and validation of cluster analysis. The principal component analysis (PCA) exhibited the spital distribution of all samples in two-dimensional space was shown by the principal component analysis (PCA).

### Survival Analysis

Cox regression and Kaplan-Meier analysis were performed by the *survival* package. The *survminer* package determined the optimal cutoff value. Univariate and multivariate analyses were utilized to calculate the hazard ratio (HR) and test the independent significance, respectively.

### Gene Set Enrichment Analysis

To further explore the biological functions of different groups, gene set enrichment analysis (GSEA) was adopted, a computational approach that tests whether the specific genes are enriched in prior gene sets. This random permutation was executed 1000 times, and gene terms with Benjamini–Hochberg (BH) corrected false discovery rate (FDR) < 0.05 were considered statistically significant. Moreover, as previously reported (Liu et al., 2021d), the single-sample gene set enrichment analysis (ssGSEA) quantified the relative abundance of 28 immune cells. Gene sets for labeling these 28 cells were generated from Charoentong et al. study (Charoentong et al., 2017). In addition, we use another algorithm (microenvironment Cell Populations-counter, MCP-counter) to calculate the immune cell infiltration (Becht et al., 2016).

### Nearest Template Prediction

To further validate our clusters in different datasets derived from distinct platforms, we performed the nearest template prediction (NTP) in each cohort. The NTP approach conveys a convenient way to compute the prediction confidence of a single patient based on gene expression data (Hoshida, 2010). In line with previous reports, we excluded samples according to the BH corrected FDR >0.2 (Isella et al., 2017; Wang et al., 2021).

### Statistical Analysis

The differential analysis between the two groups was calculated by the *limma* package. The *clusterProfiler* package was applied to perform the GSEA analysis (*via GSEA* function) and GO and KEGG Hypergeometric test (*via enrichGO* and *enrichKEGG* functions). The *glmnet* package was utilized to fit the least absolute shrinkage and selection operator (LASSO) model. The concordance index (C-index) was calculated by survival package. The *timeROC* package was utilized to estimate the receiver operating characteristic curve (ROC) and area under the ROC curve (AUC). According to the C-index estimation, the compareC package was used to determine the significance between the two variables. The ESTIMATE package assessed tumor purity. The genomic analysis was performed as previously reported (Liu et al., 2021e). All data processing, visualization, and statistical analysis were performed in the R v4.1 software.

## Results

### Proliferation Pathways Upregulated and Correlated With Worse Prognosis

**FIGURE 1:**
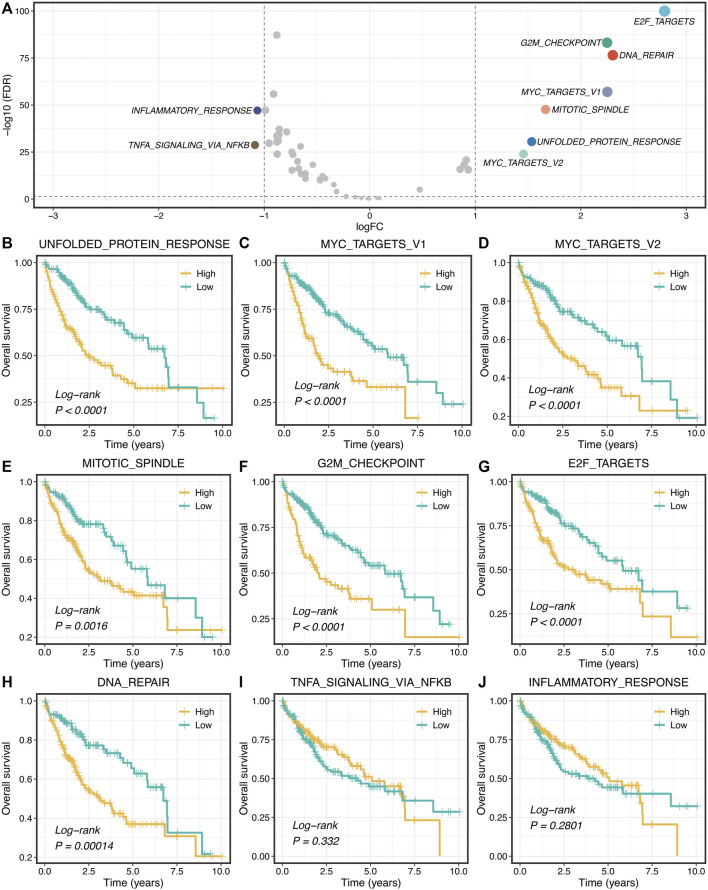
Proliferation pathways upregulated and correlated with a worse prognosis. **(A)** Differential analysis of the activity variation of fifty Hallmark gene sets between tumor and normal samples. **(B–J)** Kaplan-Meier plots of OS based on the activities of nine pathways.

Based on the 50 Hallmark gene sets, we quantified each pathway activity across the TCGA-LIHC tumor and adjacent normal samples *via* the GSVA algorithm. Subsequently, the *limma* package was utilized to identify the dysregulated pathways in HCC. As illustrated in Figure 1A, the pathway activities of two immune pathways, including inflammatory response and TNF-α signaling *via* NFKB, were weakened in HCC (Supplementary Figure S1), which suggested that immune inactivation might be a key signal of HCC progression. Interestingly, we found that all the upregulated pathways were associated with tumor proliferation, such as unfolded protein response, DNA repair, and mitotic spindle (Figure 1A and Supplementary Figure S1). Furthermore, the Kaplan-Meier survival analysis was used to evaluate the prognostic value of these dysregulated pathways. In seven proliferation pathways, patients with higher pathway activities presented significantly dismal overall survival (OS) (log-rank *p* < 0.005, Figures 1B–H). Nevertheless, two immune pathways didn’t show conspicuous prognostic significance in HCC (log-rank *p* > 0.05, Figures 1I,J). Univariate Cox regression analysis further validated these results (Supplementary Figure S2).

### Identification of Two Heterogeneous Proliferation-Related Subtypes

**FIGURE 2:**
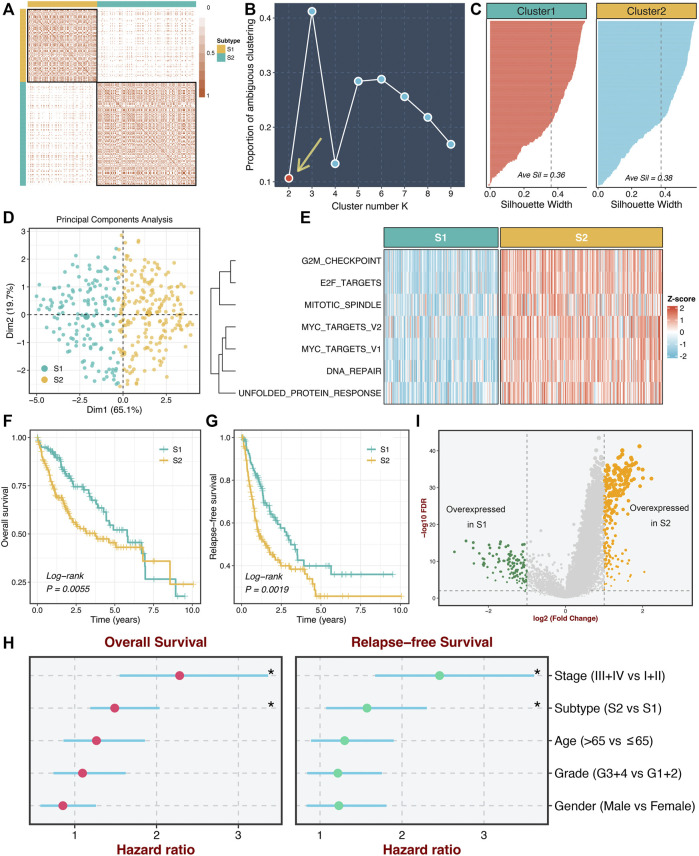
Identification of two heterogeneous proliferation-related subtypes. **(A)** The consensus score matrix of all samples when k = 2. Consistent scores between the two samples were positively correlated with the probability that they were grouped into the same cluster in different iterations. **(B)** The proportion of ambiguous clustering (PAC) score (k = 2 was the most suitable k value). **(C)** The silhouette statistic of the high- and low-risk groups. **(D)** The principal component analysis (PCA) algorithm showed the two-dimension spatial distribution of the high- and low-risk groups were shown by the principal component analysis (PCA) algorithm. **(E)** Heatmap displayed that S2 possessed a significantly higher proliferative quantity compared to S1. **(F,G)** The Kaplan-Meier plots of OS **(F)** and RFS **(G)** of the high- and low-risk groups. **(H)** Multivariate Cox regression of OS and RFS. *p < 0.05. **(I)** Differentially expressed analysis of lncRNAs between two subtypes in the TCGA-LIHC cohort.

According to the activities of seven proliferation pathways, we intended to decipher heterogeneous subtypes using consensus clustering. The consensus values range from 0 to 1 (never clustered together to always clustered together) colored by white to red in the consensus matrix (Figure 2A). When the cluster number was equal to 2, almost no color overlap in the consensus matrix (Figure 2A). We calculated the PAC of all cluster options based on the CDF curves (Supplementary Figure S3). As illustrated in Figure 2B, the two subtypes have the lowest value, indicating two optimal cluster selections.

Moreover, we further employed the silhouette statistic to confirm the stability of the clustering, and the two clusters displayed stable and robust silhouette widths (Figure 2C). To elevate the purity and significance of two subtypes, we applied silhouette width to screen out those samples residing on the periphery of the subtypes, as previously reported (Isella et al., 2017). The PCA distribution exhibited essentially no overlap between two subtypes in two-dimensional spatial space (Figure 2D). Subsequently, we quantified the proliferation pathway activities between two subtypes. As illustrated in Figure 2E, subtype 2 (S2) displayed a significantly higher abundance of seven proliferation pathways relative to subtype 1 (S1), which suggested an enhanced proliferative activity in S2. Thus, we defined S2 as hyperproliferative HCC, whereas S1 was defined as hypoproliferative HCC. In line with the proliferative state, S2 presented significantly worse OS and RFS than S1 (log-rank *p* < 0.01, Figures 2F,G). Further multivariate Cox regression analysis revealed that the proliferation-related subtypes were independent prognostic factors for OS and RFS after adjusting for clinical features (age, AJCC stage et al.) (Figure 2H). Additionally, we performed differentially expressed analysis between two subtypes based on the lncRNA expression matrix and identified 375 differentially expressed lncRNAs (DElncs), which were regarded to be associated with the proliferative activity of HCC (Figure 2I).

### Underlying Biological Processes of Proliferation-Related Subtypes

**FIGURE 3:**
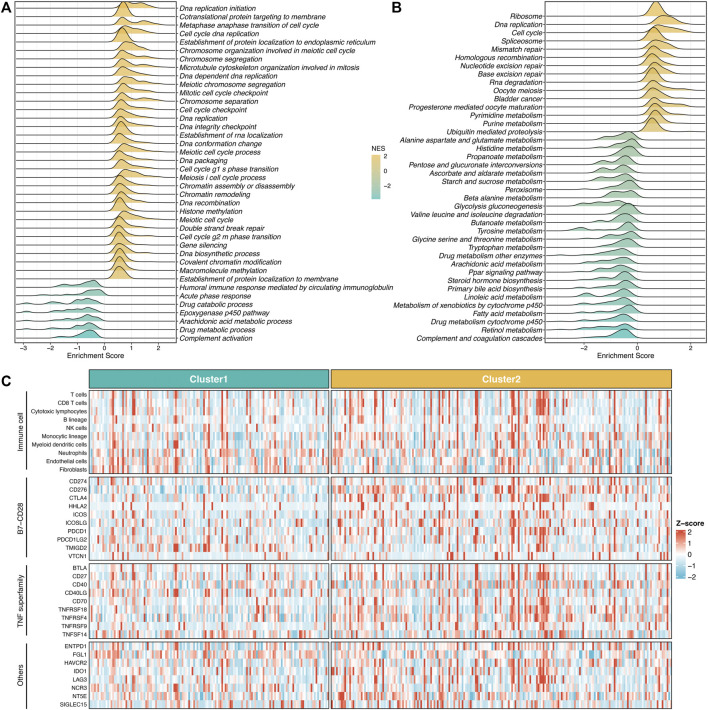
Underlying biological processes of proliferation-related subtypes. **(A,B)** GO **(A)** and KEGG **(B)** of GSEA according to the proliferative subtypes. **(C)** The immune correlation analysis between the two groups, including immune cell infiltrations and immune checkpoint.

To further explore the underlying mechanisms and biological processes of proliferation-related subtypes, we performed GSEA-GO and GSEA-KEGG analysis. As shown in Figures 3A,B, S2 enriched many pathways consistent with its inherent properties, such as DNA replication initiation, cell cycle checkpoint, metaphase anaphase transition of the cell cycle, ribosome, spliceosome, and mismatch repair. On the other hand, S1 was significantly associated with metabolism-related pathways, such as epoxygenase P450 pathway, drug catabolic process, linoleic acid metabolism arachidonic, acid metabolic process. This suggested that S1 might serve as a hypoproliferative and high-metabolic (especially lipid metabolism) subtype. Furthermore, we delineated the immune microenvironment of two subtypes. Overall, these two subtypes demonstrated less differentiated microenvironmental cell infiltration (Figure 3C and Supplementary Figure S4A). S1 had a dramatically higher abundance of fibroblasts, whereas S2 displayed a superior score in the monocytic lineage (Supplementary Figure S4A). Interestingly, the two subtypes presented striking differences in the immune checkpoint profiles. S1 was characterized by FGL1 and TMIGD2 overexpression, while S2 enriched CD276, CTLA4, ENTPD1, HHLA2, ICOS, ICOSLG, IDO1, PDCD1, TNFRSF4, TNFRSF9, and TNFRSF18 (Figure 3C and Supplementary Figure S4B). These results indicated that S2 might have more potential to benefit from immunotherapy.

### Validation of Proliferation-Related Subtypes

**FIGURE 4:**
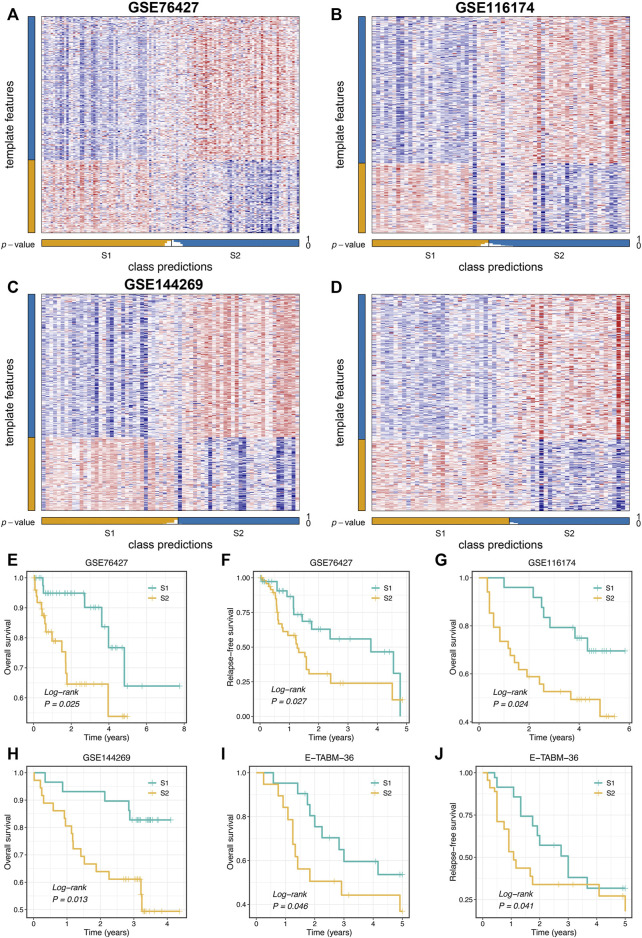
Validation of proliferation-related subtypes. **(A–D)** Heatmap of the expression level of the template feature between two subtypes in the GSE76427 **(A)**, GSE116174 **(B)**, GSE144269 **(C)**, and E-TABM-36 **(D)** datasets, respectively. **(E,F)** Kaplan-Meier curves of OS **(E)** and RFS **(F)** between the two subtypes in GSE76427. **(G,H)** Kaplan-Meier curves of OS between the two subtypes in GSE116174 **(G)** and GSE144269 **(H)**. **(I,J)** Kaplan-Meier curves of OS **(I)** and RFS **(J)** between the two subtypes in E-TABM-36.

To further validate the stability and robustness of proliferation-related subtypes in different datasets derived from distinct platforms, we performed NTP analysis in four cohorts, including GSE76427, GSE116174, GSE144269, and E-TABM-36. This algorithm could quantify the prediction confidence of a single patient based on gene expression data (Hoshida, 2010). Consistent with previous reports (Isella et al., 2017; Wang et al., 2021), we excluded samples with the BH corrected FDR >0.2. The predictive results showed high reproducibility of two subtypes in four cohorts. Moreover, the proportions of the two subtypes were quite similar in four cohorts (Figures 4A–D). We further evaluated the prognostic significance of the two subtypes. In line with the previous results, S2 displayed significantly adverse OS and RFS relative to S1 in four cohorts (log-rank *p* < 0.05, Figures 4E–H). Taken together, the two proliferation-related subtypes were reproducible and stable in HCC.

### ProLnc: Recognizing the Latent LncRNA Modulators of Proliferation Pathways

**FIGURE 5:**
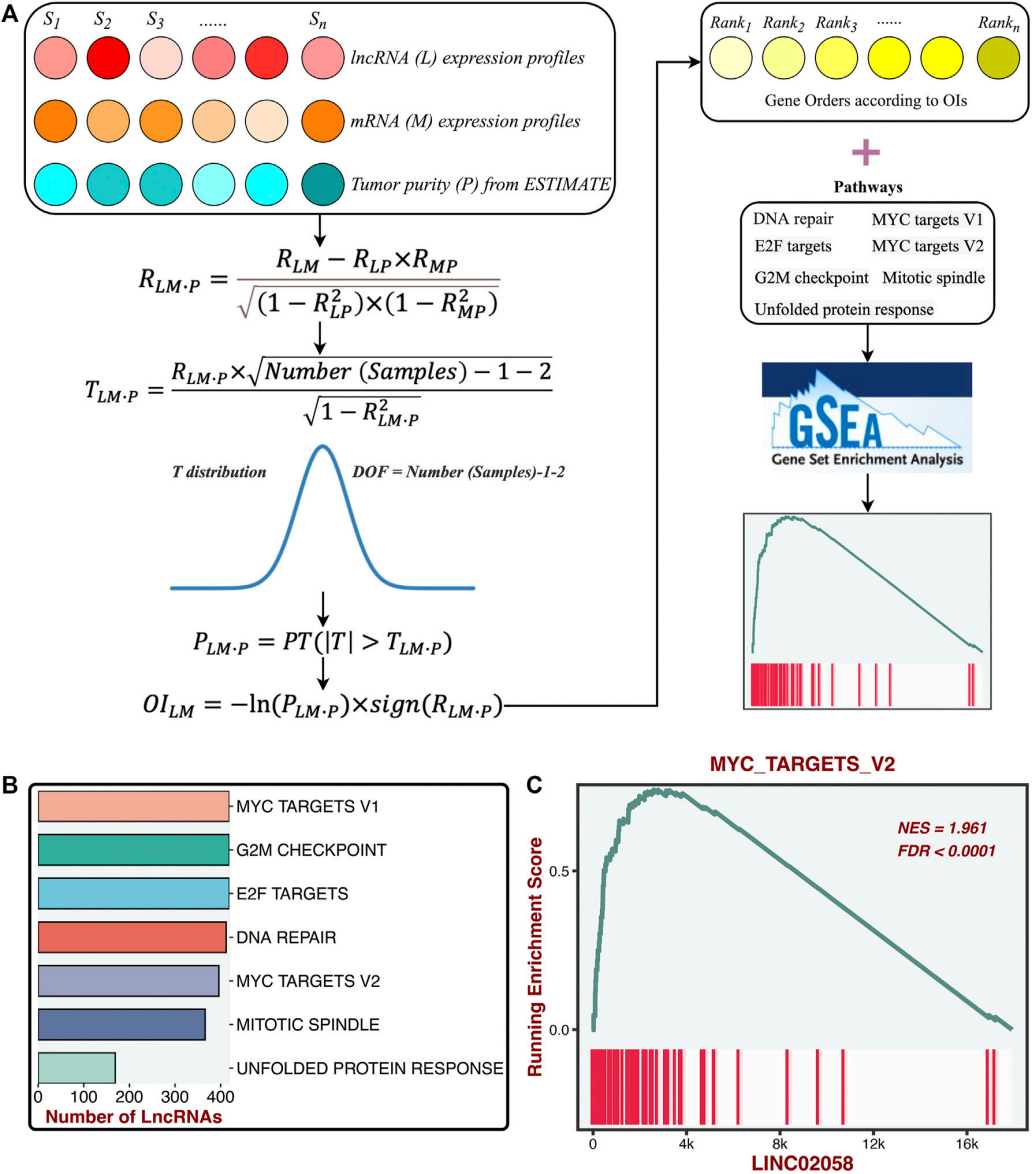
Identification of the latent lncRNA modulators of proliferation pathways. **(A)** ProLnc: recognizing the latent lncRNA modulators of proliferation pathways. **(B)** The number of lncRNAs significantly associated with proliferation pathways. **(C)** LINC02058 was dramatically associated with MYC targets V2.

To further determine the potential lncRNA modulators of proliferation pathways, we constructed a compositive pipeline that relied on the previous reports (Li et al., 2020; Liu et al., 2021f) (Figure 5A). We first calculated its first-order partial correlation coefficients (PCCs) with all mRNAs adjusted for tumor purity for each lncRNA. Based on PCC and degree of freedom (DOF), we calculated the T statistic and further measured the *p*-value. Subsequently, mRNA’s ordered index (OI) was calculated from *p*-value and PCC. According to the descending order of all OIs, all mRNAs were sorted into a gene rank list. The specific lncRNA gene rank list was subjected to the GSEA algorithm to test the correlations between this lncRNA and seven proliferation pathways. Ultimately, the enrichment score (ES) and FDR of a specific lncRNA were generated from the GSEA pipeline and then converted into a final significance index (FSI), as follows:

FSI=(1−2×FDR)×sign(ES)

*sign* function was utilized to extract the plus and minus of ES. The lncRNAs with the |FSI| and FDR <0.001 were regarded as the proliferation-derived lncRNAs. In total, we identified 474 lncRNA modulators of seven proliferation pathways (Figure 5B and Supplementary Table S1). For instance, LINC02058 was dramatically associated with MYC targets V2 (Figure 5C).

### Identifying the Stable Prognostic LncRNAs *via* Bootstrapping

**FIGURE 6:**
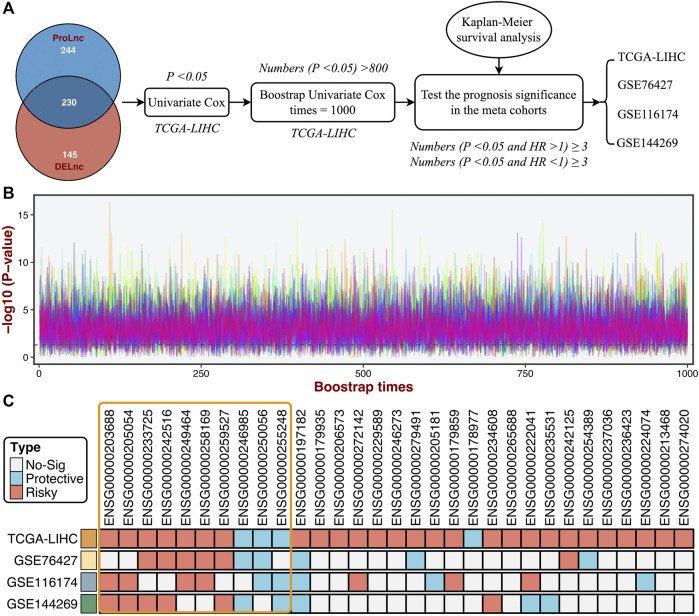
Identifying the stable prognostic lncRNAs *via* bootstrapping univariate Cox regression analysis. **(A)** A multi-step pipeline to determine the regular prognostic PALs (SPPALs). **(B)** The bootstrapping univariate Cox regression analysis was repeated 1000 times, and 31 lncRNAs incorporated in 80% of resampling runs (*p* < 0.05) were kept for subsequent analysis. **(C)** The prognostic significance of 31 lncRNAs in four cohorts.

Based on the DElncs and the lncRNAs from the ProLnc framework, we determined a total of 230 proliferation-associated lncRNAs (PALs) (Figure 6A). We developed a multi-step pipeline to identify further the stable prognostic PALs (SPPALs) (Figure 6A). First of all, preliminary screening was performed to include prognosis-related lncRNA in TCGA-LIHC cohort *via* univariate Cox regression analysis, where lncRNAs with *p*-value < 0.01 were selected for further research. Next, we use bootstrapping to test the lncRNAs which passed initial filtering for robustness. We extracted 70% samples randomly and performed univariate Cox regression on these samples to assess the correlation between the lncRNA expression and prognosis. After 1000 times calculations of this procedure, and the lncRNAs incorporated in 80% of resampling runs (achieved *p* < 0.05 in robustness testing) were kept for subsequent analysis. 31 PALs were determined (Figure 6B and Supplementary Table S2). To further test the prognostic compatibility of these PALs on different platforms, we explored their prognostic significance in four cohorts, including TCGA-LIHC, GSE76427, GSE116174, and GSE144269 (Figure 6C), E-TABM-36 was discarded due to its lack of lncRNAs. As displayed in Figure 6C, PALs that were significant in three or four cohorts were considered SPPALs. Notably, lncRNAs were excluded if they showed opposite effects in different cohorts (e.g., *ENSG00000197182*). Therefore, we identified 10 SPPALs that stably correlated with prognosis, seven were risk factors, and three were protective factors (Figure 6C).

### Development of Prognostic Signature

**FIGURE 7:**
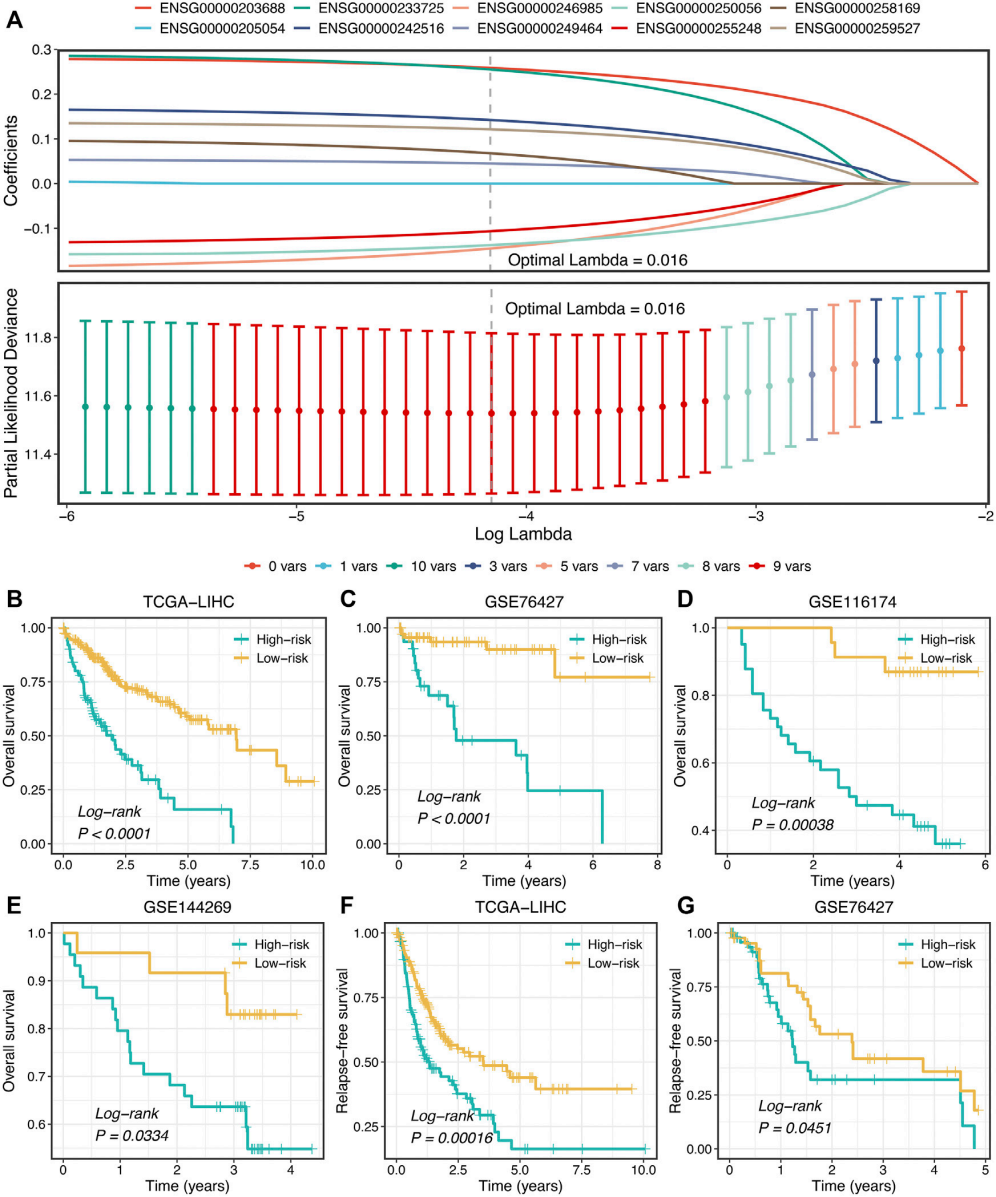
Development of prognostic signature. **(A)** LASSO coefficient profiles of 10 lncRNAs and partial likelihood deviance for LASSO coefficient profiles. **(B–E)** Kaplan-Meier curves of OS according to ProLncS in TCGA-LIHC **(B)**, GSE76427 **(C)**, GSE116174 **(D)**, and GSE144269 **(E)**. **(F,G)** Kaplan-Meier curves of RFS between the two groups in TCGA-LIHC **(F)** and GSE76427 **(G)**.

Subsequently, LASSO regression was further utilized to construct the model based on the expression profiles of 10 SPPALs. The LASSO algorithm is a popular approach for regression with high-dimensional predictors. It introduces a penalty parameter lambda to shrink regression coefficients, enhancing the generalization ability of the model and preventing overfitting (Liu et al., 2021g; Liu et al., 2021h). The penalty parameter lambda controlled the degree of shrinkage: the larger the lambda, the smaller the coefficients, even some regression coefficients were shrunk to exactly zero (Figure 7A). The 10-fold cross-validation was applied to determine the optimal lambda, and the partial likelihood deviance was calculated to evaluate the efficacy variation between the training and validation subsamples. In this way, we choose the optimal lambda = 0.016 when the partial likelihood deviance achieves the lowest value (Figure 7A). Thus, we obtained the optimal model (termed “ProLncS”) composed of nine SPPALs and calculated the risk score for each patient (Figure 7A and Supplementary Table S3). Compared with the low-risk group, patients in the high-risk group displayed worse OS (log-rank *p* < 0.05, Figures 7B–E).

Moreover, the ProLncS model remained statistically significant after controlling for available clinical traits, including AJCC stage, histological grade, body mass index, alcohol, smoking, HBV, HCV, and HDV (Supplementary Figures S5A–D). This indicated that ProLncS was an independent factor for OS. To further assess the correlations between ProLncS and relapse, we explored the significance of ProLncS for RFS in TCGA-LIHC and GSE76427 datasets with relapse information. As shown in Figures 7F,G, RFS was also more dismal in the high-risk group than the low-risk group (log-rank *p* < 0.05). Likewise, ProLncS also independently predict the RFS in TCGA-LIHC and GSE76427 datasets (Supplementary Figures S6A,B).

### Evaluation and Comparison of ProLncS

**FIGURE 8:**
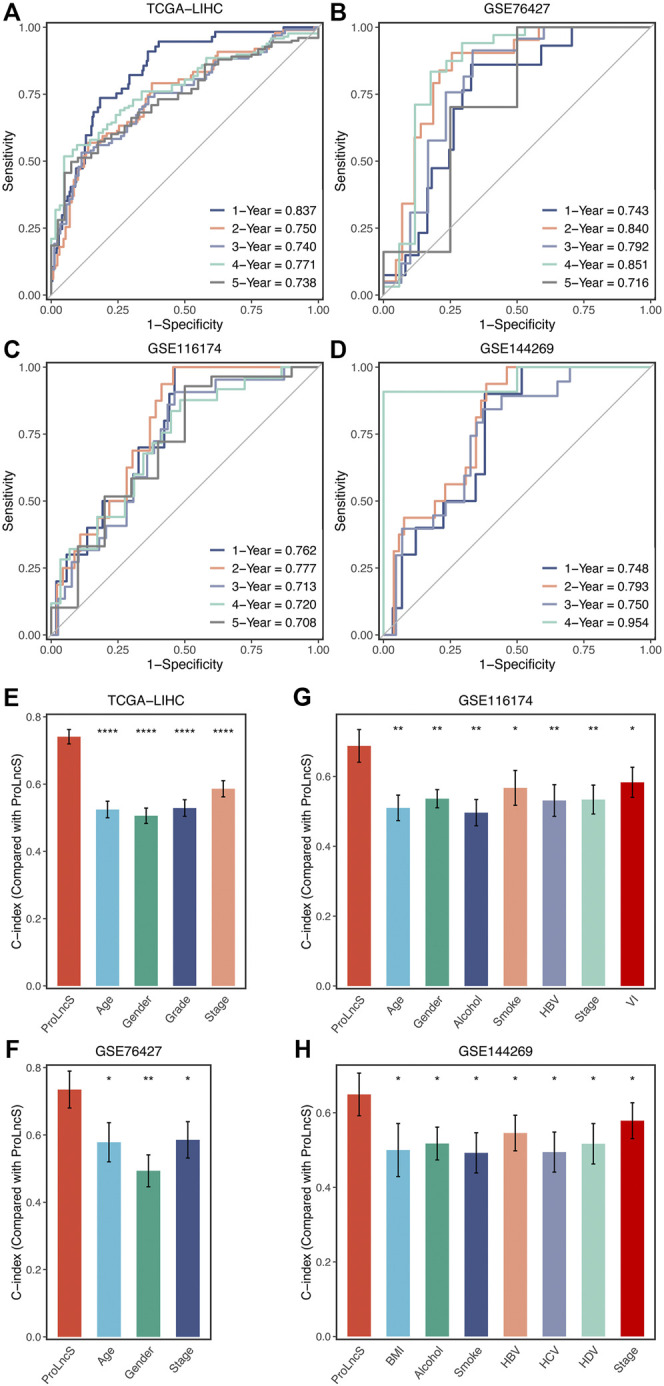
Evaluation and comparison of ProLncS. **(A–D)** Time-dependent ROC analysis for predicting OS at 1–5 years in the TCGA-LIHC **(A)**, GSE76427 **(B)**, GSE116174 **(C)**, and GSE144269 **(D)**. The ability of ProLncS to predict prognosis of HCC was compared with common clinical variables in the TCGA-LIHC **(E)**, GSE76427 **(F)**, GSE116174 **(G)**, and GSE144269 **(H)**. *p < 0.05, **p < 0.01, ****p < 0.0001.

In this study, the time-dependent ROC and C-index were utilized to assess the predictive performance of the ProLncS model. The 1-, 2-, 3-, 4-, and 5-years AUCs were 0.837, 0.750, 0.740, 0.771, and 0.738 in TCGA-LIHC, 0.743, 0.840, 0.792, 0.851, and 0.716 in GSE76427, and 0.762, 0.777, 0.713, 0.720, and 0.708 in GSE116174 (Figures 8A–C). Since the follow-up time of GSE144269 did not exceed 5 years, we only calculated AUCs of 1–4 years, which were 0.748, 0.793, 0.750, and 0.954, respectively (Figure 8D). Additionally, the C-index of four cohorts were 0.741, 0.735, 0.687, and 0.649, respectively. Overall, the ProLncS model displayed stable and accurate performance for predicting the prognosis. To compare the performance of ProLncS with traditional clinical traits, the *compareC* package was used to determine the significance between two variables according to the C-index estimation. Encouragingly, ProLncS presented significantly superior accuracy than these traditional clinical traits, including age, gender, histological grade, AJCC stage, body mass index, alcohol, smoking, HBV, HCV, and HDV (Figures 8E–H). In addition, we collected previously published models of HCC (Supplementary Table S4) to compare the prediction prognosis ability of ProLncS and these models. As shown in Supplementary Figure S7, ProLncS presented significantly superior accuracy than these models.

### Genomics Landscape of ProLncS

**FIGURE 9:**
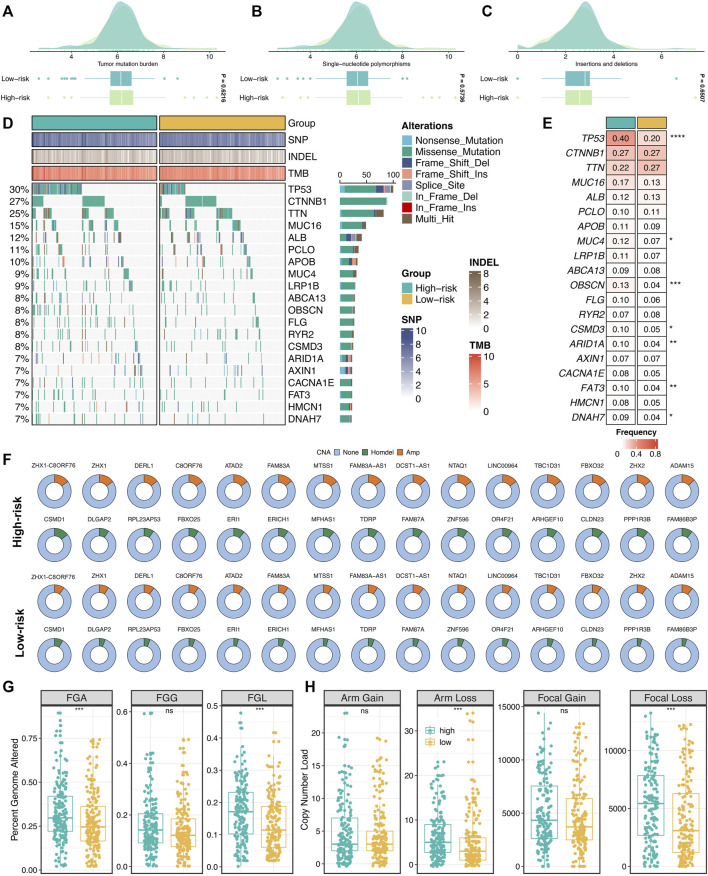
Genomic landscape of ProLncS. **(A–C)** Distributions of TMB **(A)**, SNP **(B)**, and Indel **(C)** between two risk groups. **(D,E)** Mutational landscape **(D)** and frequency **(E)** of the top 20 FMGs between two groups. **(F)** CNV frequency of 30 FAGs/FHGs between two groups. **(G)** Distributions of a fraction of genome alteration, gain and lost between two groups. **(H)** Distributions of arm gain, arm loss, focal gain, and focal loss. ^ns^ *P* <0.05, *p < 0.05, **p < 0.01, ***p < 0.001, ****p < 0.0001.

Furthermore, we explored the genomics landscape of ProLncS as previously reported (Liu et al., 2021e). As displayed in Figures 9A–C, there were no significant differences in Single-nucleotide polymorphism (SNP), tumor mutation burden (TMB), and deletion or insertion (Indel), between the high- and low-risk groups. To get deep insights, we carved the portrait of 20 frequently mutated genes (FMGs) between the two groups (Figure 9D). Intriguingly, the two groups displayed significantly mutational differences in these FMGs (Figure 9E). The high-risk group patients possessed more mutations of TP53, MUC4, OBSCN, CSMD3, ARID1A, FAT3, and DNAH7 than the low-risk group patients (Figure 9E). Subsequently, we characterized the copy number variation (CNV) status of 30 frequently amplification genes (FAGs)/frequently homozygous deletion genes (FHGs) between the two groups (Figure 9F). Overall, the high-risk group also displayed superior variations in CNV, especially the cytobands 8p23-8p24 (Supplementary Table S5). For example, compared with the low-risk group patients, the high-risk group patients showed more amplification of ATAD2, C8ORF76, FAM83A-AS1, FBXO32, LINC00964, MTSS1, NTAQ1, TBC1D31, ZHX1, ZHX1-C8ORF76, and ZHX2, as well as more deletions of CLDN23, ERI1, FAM86B3P, MFHAS1, PPP1R3B, and CSMD1 (Figure 9F and Supplementary Table S5). Of note, there were no significant differences in FGG, arm gain, and focal gain (Figures 9G,H). For another, the high-risk group demonstrated superior burdens of copy number deletion at the level of chromosome arms, fragments, and bases (Figures 9G,H). Collectively, the high-risk group patients conveyed a significantly genomic instability and displayed abundant molecular alterations.

### Exploring the Potential Mechanisms of ProLncS

**FIGURE 10:**
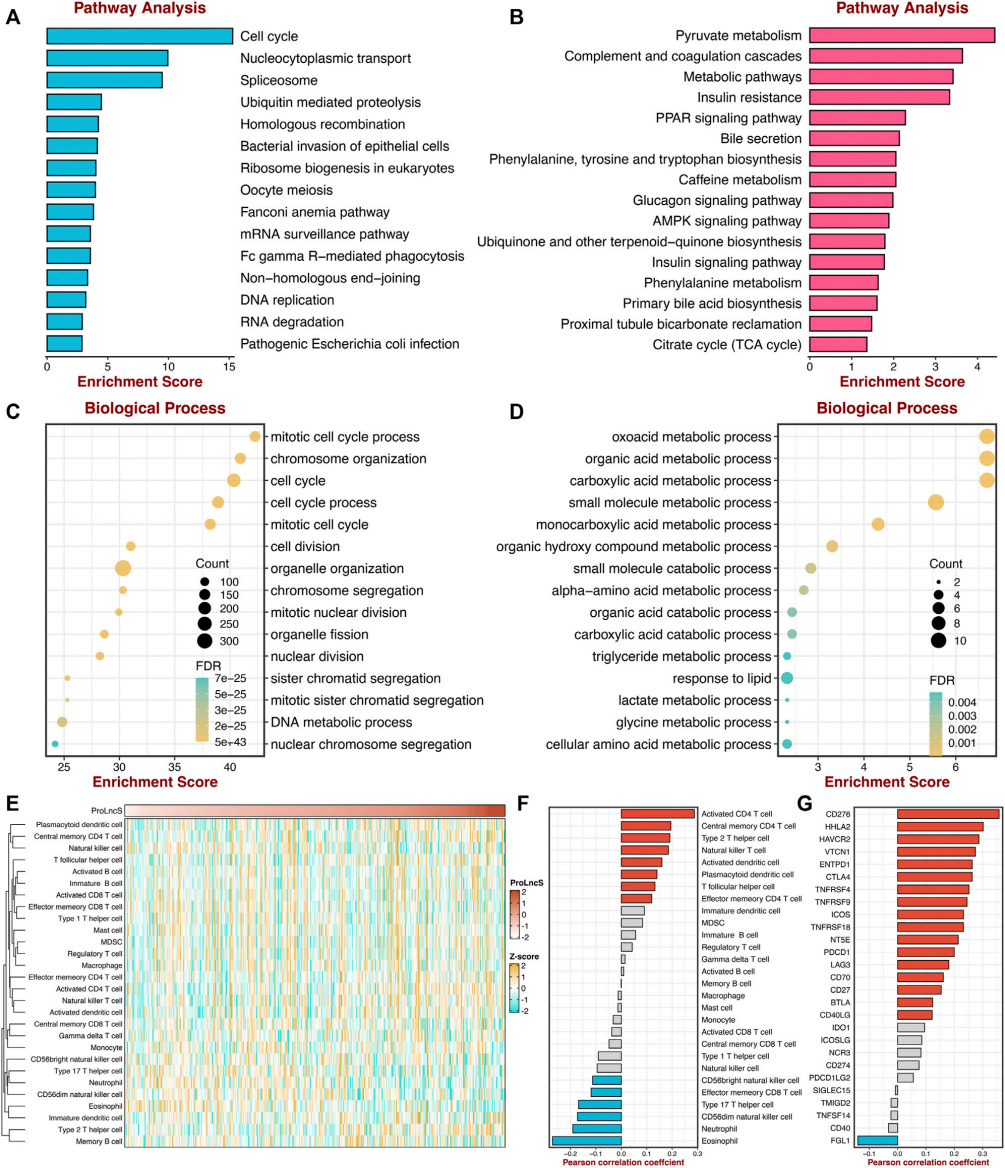
Exploring the potential mechanisms of ProLncS. **(A,B)** The KEGG pathway enrichment analysis of genes significantly correlated with ProLncS. The top 15 positive **(A)** and negative **(B)** pathways are associated with ProLncS. **(C,D)** GO enrichment analysis of the top 15 positive **(C)** and negative **(D)** biological processes. **(E)** Distribution of 28 immune cells with ProLncS. **(F)** Correlations between ProLncS with 28 immune cells. **(G)** Correlations between ProLncS with 27 immune checkpoint molecules. Red and blue bars mean *p* < 0.05, and grey bar means *p* > 0.05.

To further reveal the underlying mechanisms of ProLncS, we intended to identify the critical modulators of ProLncS. We calculated the Pearson correlation coefficients between ProLncS and all genes. Genes with |r | >0.4 and FDR <0.001 were considered to be significantly associated with ProLncS (Supplementary Figures S8A,B). Subsequently, KEGG and GO analyses were performed on these genes. As shown in Figures 10A,B, genes that positively correlated with ProLncS enriched many pathways related to proliferation, whereas genes that negatively correlated with ProLncS were significantly associated with metabolism-related pathways. The GO analysis further validated these results from KEGG (Figures 10C,D). In addition, we delineated the immune landscape of ProLncS (Figure 10E). As displayed in Figure 10F, ProLncS positively correlated with activated CD4^+^ T cell, natural killer T cell, activated dendritic cell, T follicular helper cell, and effector memory CD4 T cell, while negatively correlated with CD56 bright natural killer cell, effector memory CD8^+^ T cell, type 17 T helper cell, CD56dim natural killer cell, neutrophil, and eosinophil. This suggested that patients with high ProLncS stored more abundance of immune cells. We further characterized the immune checkpoint profiles (Liu et al., 2021i) of ProLncS, and the results also revealed predominant correlations (Supplementary Figure S9 and Figure 10G). ProLncS was positively related to CD276, HHLA2, VTCN1, ENTPD1, CTLA4, TNFRSF4, TNFRSF9, ICOS, TNFRSF18, NT5E, PDCD1, LAG3, CD70, CD27, BTLA, and CD40LG, and negatively correlated with FGL1 (Figure 10G and Supplementary Figure S9). Overall, these results suggested that immunotherapy was more suitable for patients with high ProLncS score.

## Discussion

Hallmark gene sets decrease the variation and redundancy, systematically pool key information of the initial founder sets, and provide more concise and refined inputs for gene set analysis (Liberzon et al., 2015). Based on the pathway activities assessment from the GSVA algorithm, the gene expression matrix was transformed to a gene-set activity matrix. The proliferation pathways, including unfolded protein response, G2M checkpoint, mitotic spindle, and DNA repair, were significantly upregulated and associated with worse prognosis in HCC, which indicated these pathways might play essential roles in the occurrence and progression of HCC. Therefore, in our opinion, these seven proliferation pathways were more predominant than other pathways from Hallmark in evaluating the progression and prognosis of HCC.

Thus, according to the pathway activities of these seven proliferation pathways, we decoded two heterogeneous proliferation-related subtypes using consensus clustering. S2 displayed enhanced proliferative activities and a worse prognosis, whereas S1 was defined as hypoproliferative HCC and possessed a favorable prognosis. Further multivariate Cox regression analysis demonstrated that the two subtypes could independently predict the prognosis of HCC, which thus served as a promising tool for clinical management in clinical settings. We also revealed the underlying biological processes of the two subtypes. S2 enriched proliferative pathways, and S1 was significantly associated with metabolism-related pathways. Taken together, our results suggested S1 might serve as a hypoproliferative and high-metabolic (especially lipid metabolism) subtype, while S2 was deemed as the malignant phenotype associated with the hyperproliferative HCC.

Our study also delineated the immune microenvironment of two subtypes but did not observe significantly different cell populations. In contrast, the two subtypes presented striking differences in the immune checkpoint profiles. S1 was characterized by FGL1 overexpression, while S2 enriched CD276, CTLA4, ENTPD1, HHLA2, ICOS, ICOSLG, IDO1, PDCD1, TMIGD2, TNFRSF4, TNFRSF9, and TNFRSF18. For example, CD276 belongs to B7-CD28 superfamilies and profoundly impacts the suppression of T-cell function (Picarda et al., 2016). With a preferential expression on cancer cells, CD276 could be considered an attractive target for cancer immunotherapy (Picarda et al., 2016). Also, HHLA2 was another potential target for immunotherapy due to its coinhibitory role, overexpression across solid cancers, and correlation with adverse prognosis (Wei et al., 2021). Thus, our findings suggested that S2 might have more potential to benefit from immunotherapy.

Additionally, the NTP algorithm was applied to validate further the stability and robustness of proliferation-related subtypes in different datasets derived from distinct platforms, including GSE76427, GSE116174, GSE144269, and E-TABM-36. The predictions exhibited high reproducibility of two subtypes in four cohorts. Moreover, the proportions of the two subtypes were quite similar in four cohorts. These results proved the robustness of the two subtypes and laid a foundation for identifying proliferation-related lncRNAs.

Elegant studies demonstrate that lncRNAs are significantly implicated in tumor proliferation (Klingenberg et al., 2017; DiStefano and Gerhard, 2021). In our research, we used two approaches to identify proliferation-related lncRNAs to assess the prognosis further and improve the clinical outcomes. Firstly, we screened out lncRNAs from the differential analysis between two robust subtypes. Next, we developed a second pipeline to recognize the latent lncRNA modulators of proliferation pathways. Combining two methods, we determined a total of 230 PALs. Subsequently, based on the bootstrapping univariate Cox regression analysis and the verification of multiple cohorts, we further identify 10 SPPALs that stably correlated with prognosis (three protective factors and seven risk factors). Overall, these lncRNAs might be latent biomarkers for evaluating the development and prognosis of HCC.

Importantly, subsequent work was focused on the accurate prognostic assessment *via* the LASSO machine learning algorithm. Our ProLncS signature could independently predict the prognosis of HCC. Patients with high ProLncS score displayed unfavorable OS and RFS relative to those with low ProLncS score. The assessment results of time-dependent ROC and C-index demonstrated that ProLncS afforded great accuracy in predicting the prognosis of HCC. These results appeared in four different cohorts, showing stable and robust performance. In addition, we also compared the efficiency of ProLncS with traditional clinical traits, and encouragingly, ProLncS displayed superior accuracy than these traditional clinical traits, including age, gender, histological grade, AJCC stage body mass index, alcohol, smoking, HBV, HCV, and HDV. The clinical convertibility of our ProLncS model indicated its promising potential in the clinical management of HCC.

Furthermore, we delineated the genomic landscape of ProLncS. Whether tumor progression and molecular variation occur successively or dynamically interact with each other remains to be further demonstrated (Dhanasekaran et al., 2019; Ding et al., 2019). From a global perspective, there were no significant differences in SNP, Indel, and TMB between the high- and low-risk groups. However, some driver genes of HCC were an increased expression in the high-risk group. For example, TP53, a genome keeper, regulates the cell cycle and promotes cell apoptosis or cell senescence, and its mutations could promote cell proliferation and inhibit cell apoptosis (Calderaro et al., 2019).

Additionally, the high-risk group patients also displayed higher variation in CNV, especially the cytobands 8p23-8p24. Collectively, the high-risk group patients conveyed a significantly genomic instability and showed abundant molecular alterations. Subsequently, we explored the potential mechanisms of ProLncS. KEGG and GO analyses were performed on genes significantly associated with ProLncS. The positively related genes enriched plenty of proliferation-related pathways, whereas negatively associated genes were significantly related to metabolism-related pathways. Moreover, we also found ProLncS positively correlated with multiple immune cells and abundant immune checkpoint molecules. Taken together, these results suggested that immunotherapy was more suitable for patients with high ProLncS score, which might further improve clinical outcomes in high-risk patients.

As far as we know, this is the first study to date identifying the proliferation-related lncRNAs based on Hallmark pathways. Despite the ProLncS model being promising, some limitations should be acknowledged. First, all HCC tissues included in our study were retrospective, and future research should be carried out to verify the results. Second, our study was based on a public database, and further clinical trials should be executed. Third, due to the data heterogeneity of different platforms, we only included the intersection lncRNAs, and plenty of latent lncRNAs may have been excluded or not detected.

## Conclusion

In conclusion, this study established the comprehensive links between tumor proliferation and lncRNAs in HCC. We proposed and validated a novel proliferation-related classification system, presenting distinct proliferative states, risk stratification, and underlying mechanisms. Combining with the DElncs from two subtypes and lncRNAs from our integrated framework (ProLnc), we determined the potential lncRNAs modulators of the proliferation pathway. Subsequently, our study developed and verified a feasible and reproducible proliferation-related lncRNA signature, which afforded a stable and accurate efficacy in evaluating the prognosis of HCC. Collectively, our ProLncS could serve as a promising tool to further optimize decision-making in the clinical management of HCC.

## Data Availability

The datasets presented in this study can be found in online repositories. The names of the repository/repositories and accession number(s) can be found in the article/Supplementary Material.
