# Supplementary material for: Title: Multi-Omics and Immune Landscape of Proliferative LncRNA Signatures: Implications for Risk Stratification and Immunotherapy in Hepatocellular Carcinoma
Source: Front Pharmacol. 2022 May 18;13:907433. doi: 10.3389/fphar.2022.907433 (PMC9158467; doi:10.3389/fphar.2022.907433)
Supplement: Supplementary file 1 [file DataSheet1.docx]

**Supplementary Figures**

- Figure S1
- Figure S2
- Figure S3
- Figure S4
- Figure S5
- Figure S6
- Figure S7
- Figure S8
- Figure S9


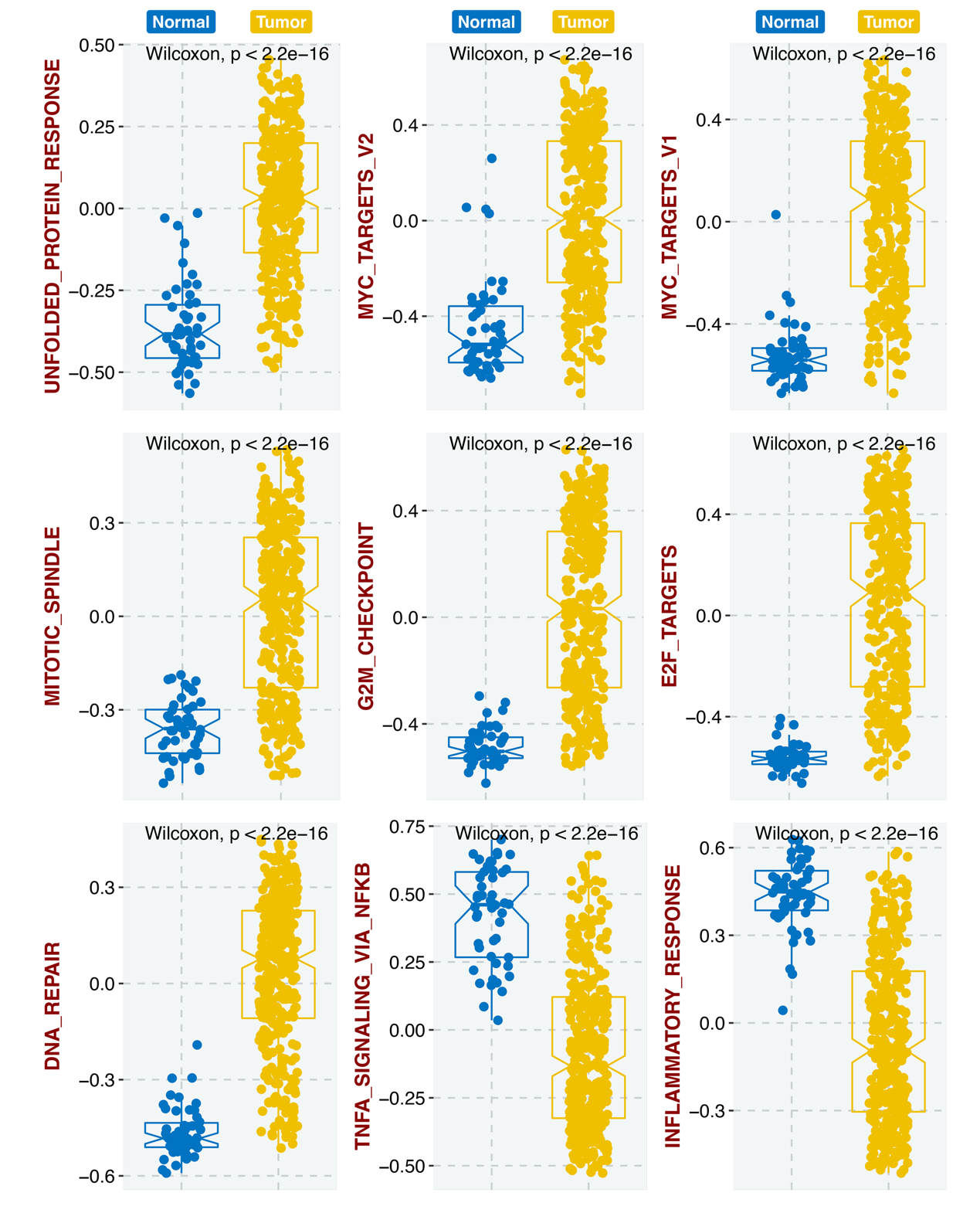


**Figure S1.** Distribution of nine pathway activities between normal and tumor tissues.


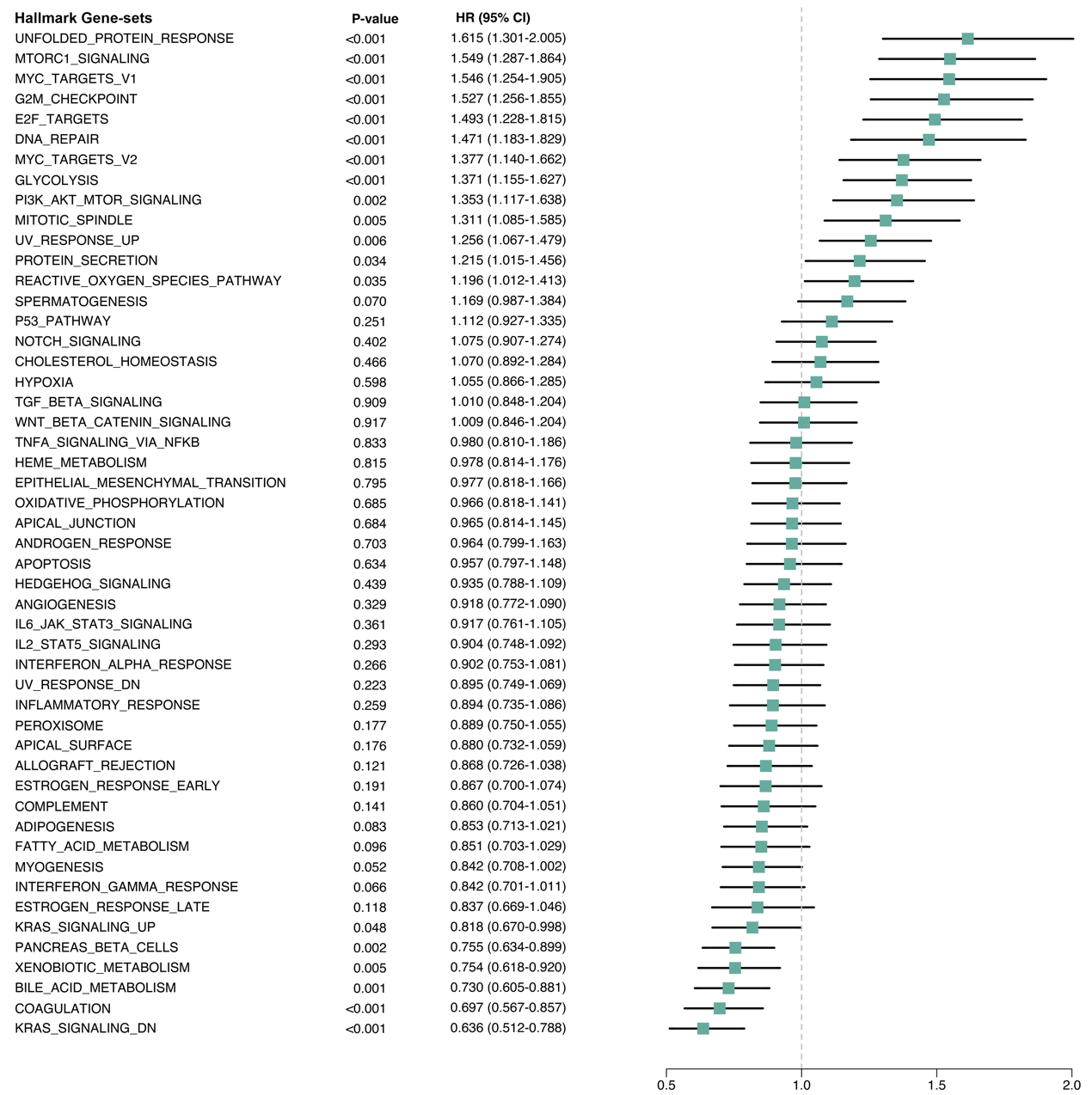


**Figure S2**. Univariate Cox regression analysis of fifty Hallmark gene sets in HCC.


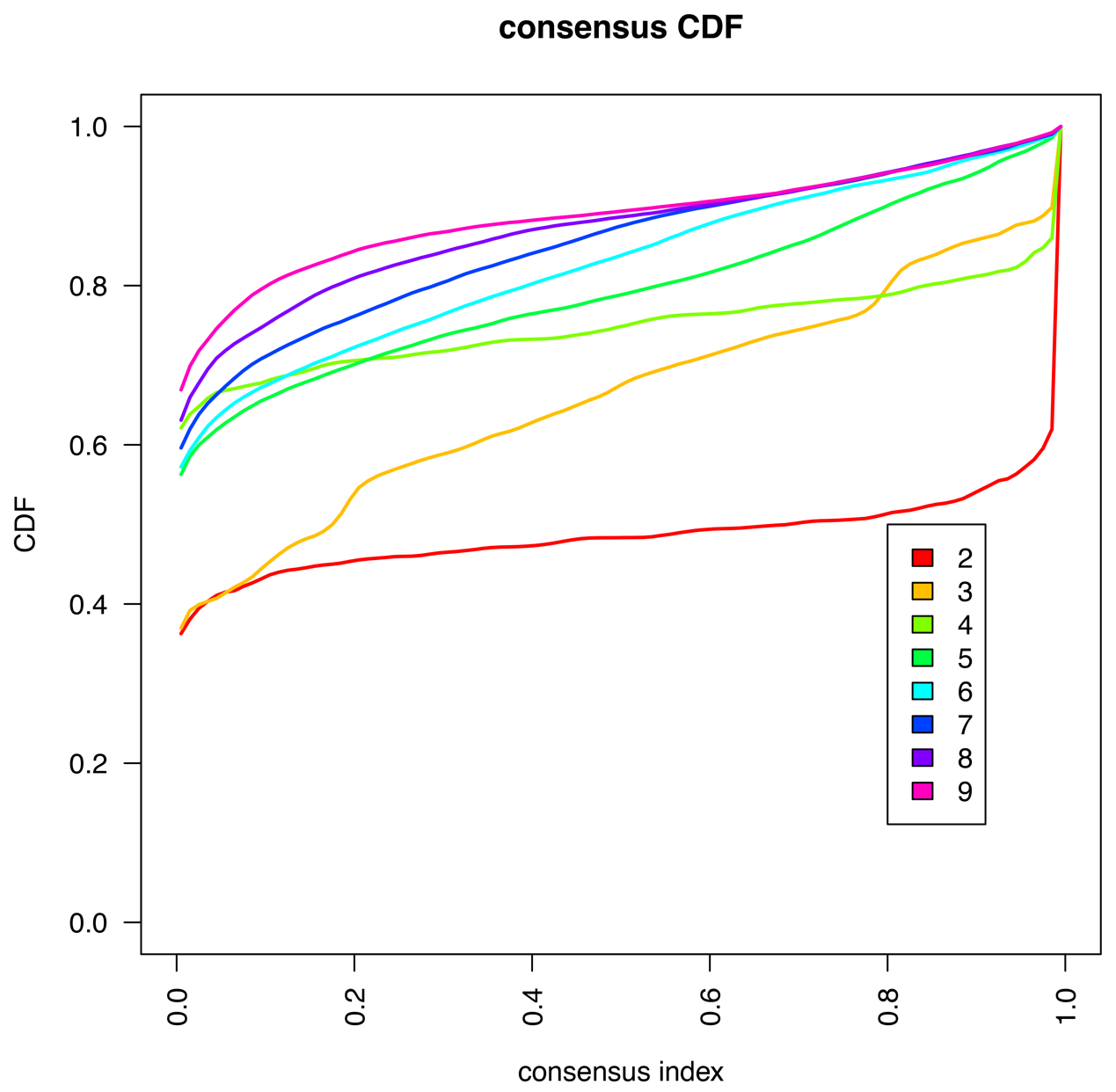


**Figure S3**. The CDF curves of consensus matrix for each k (indicated by colors).


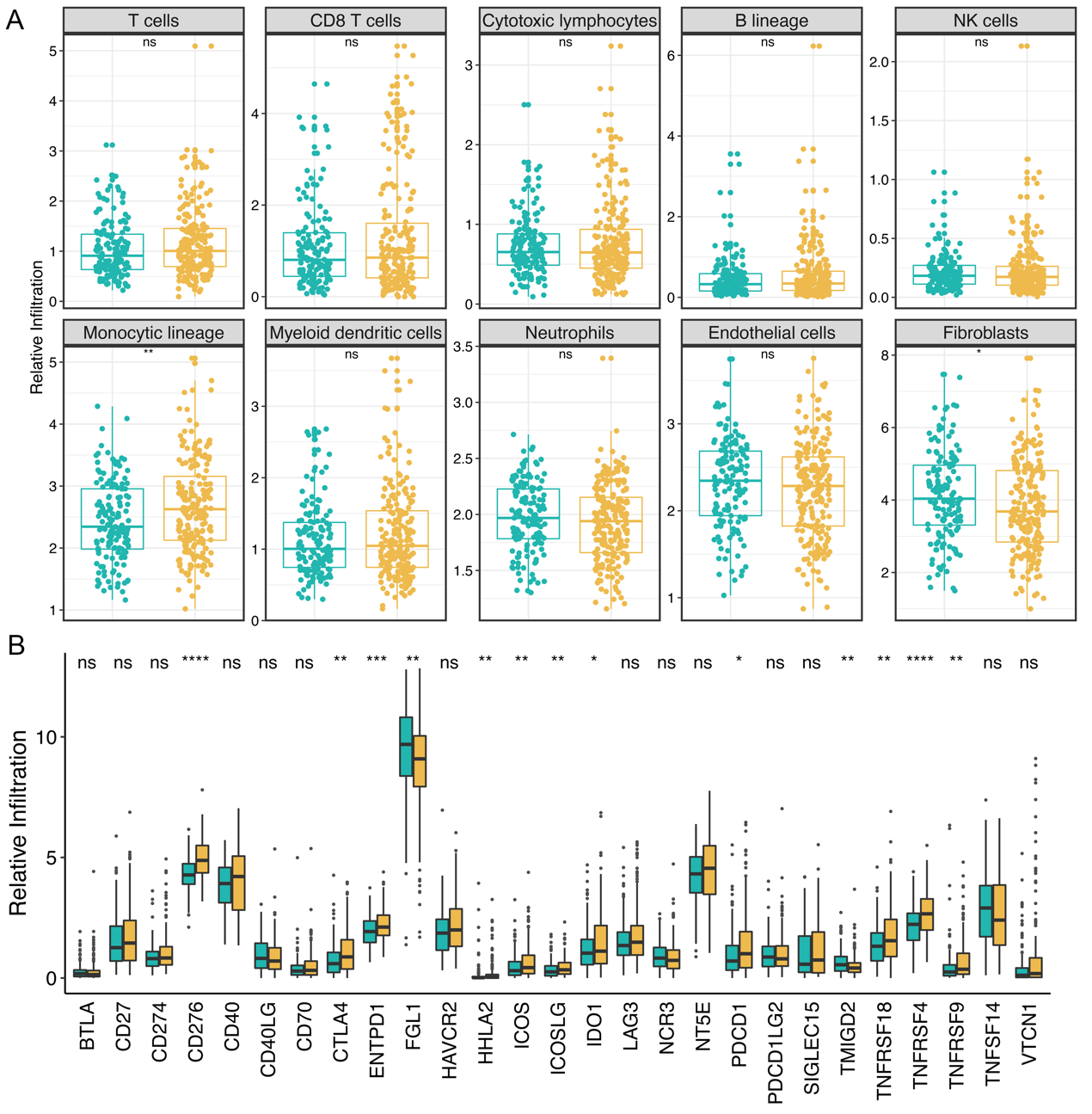


**Figure S4.** Distinct microenvironment patterns between two subtypes. (**A**) Distributions of 10 immune and stromal cells between two clusters. (**B**) Distributions of 27 immune checkpoint molecules between two clusters. ^ns^*P* >0.05, **P* <0.05, ***P* <0.01, ****P* <0.001, *****P* <0.0001.


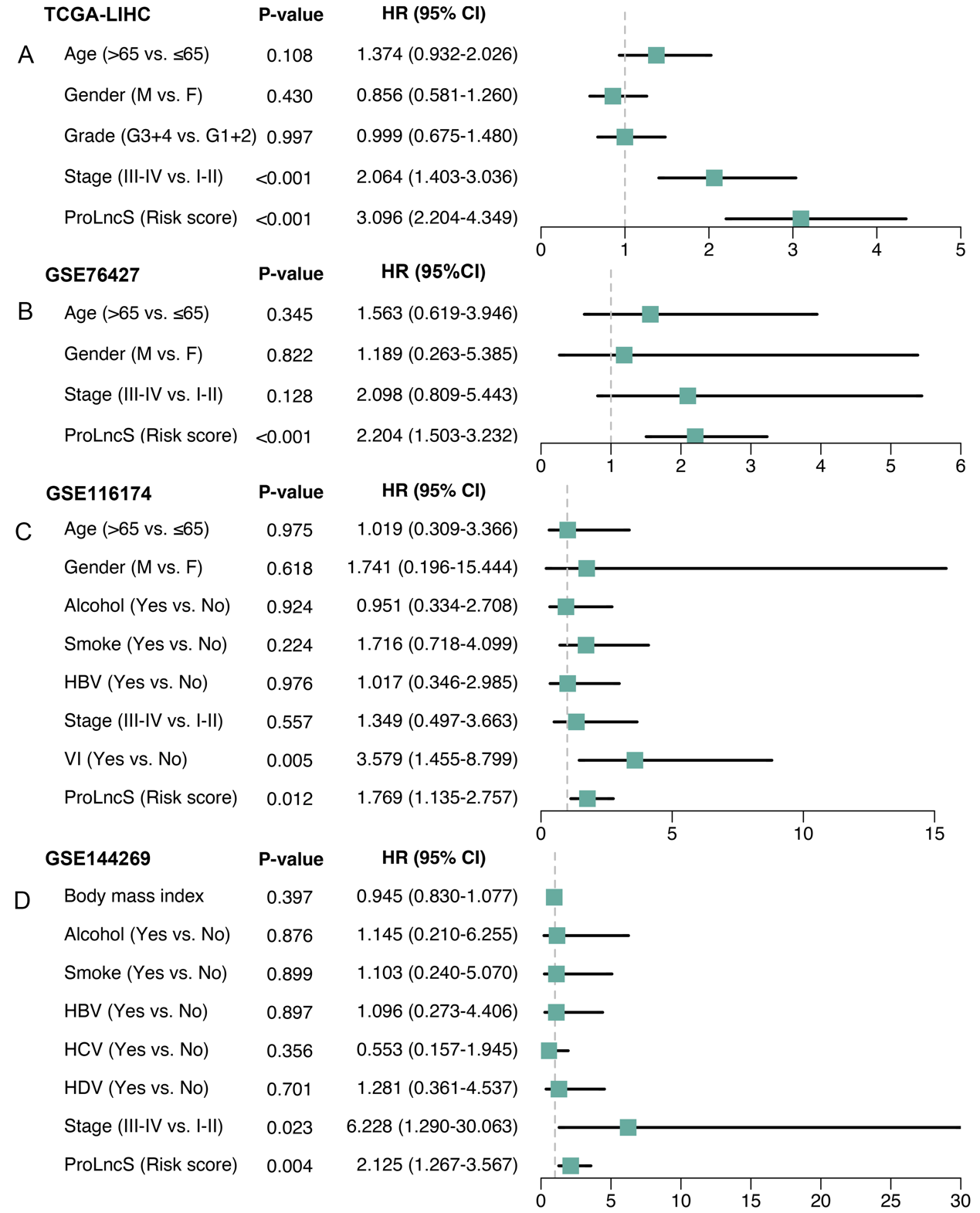


**Figure S5.** Multivariate Cox regression analysis of OS in TCGA-LIHC (**A**), GSE76427 (**B**), GSE116174 (**C**), and GSE144269 (**D**).


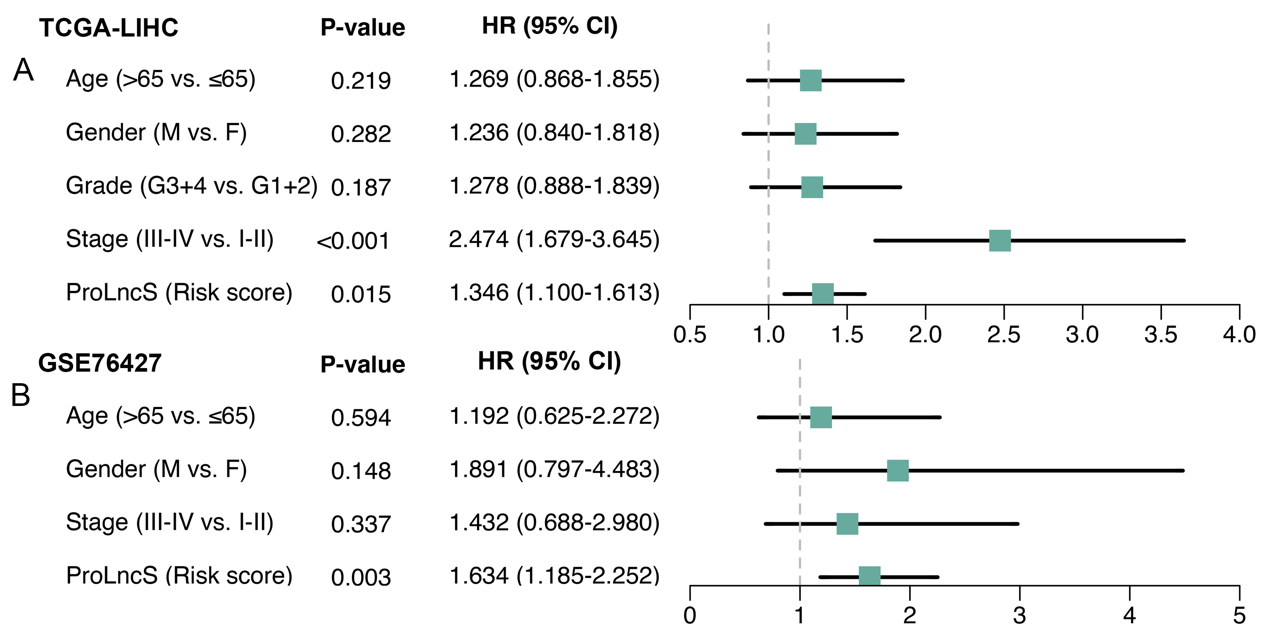


**Figure S6.** Multivariate Cox regression analysis of RFS in TCGA-LIHC (**A**) and GSE76427 (**B**).


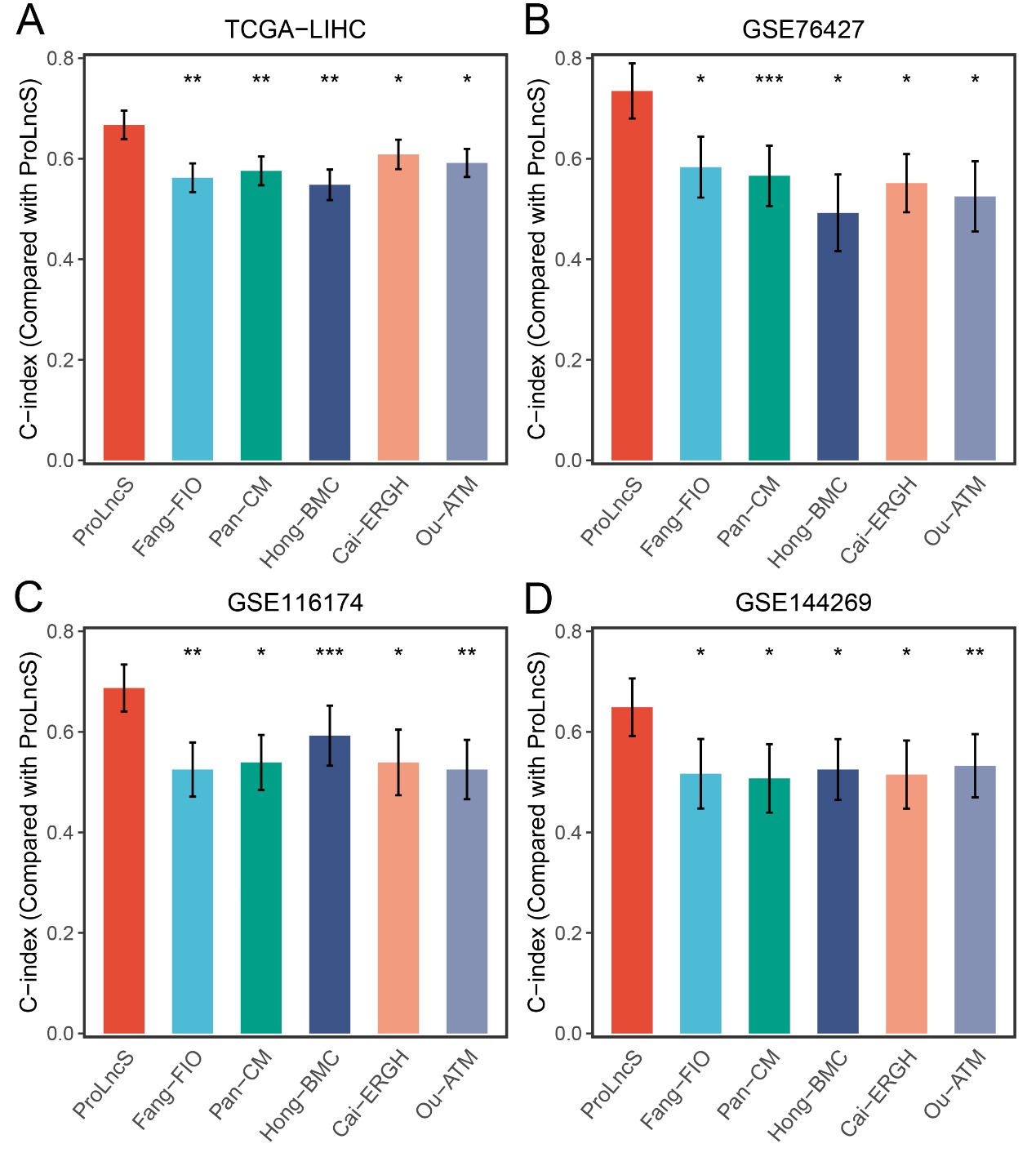


**Figure S7. Comparison between ProLncS** **and other models.** The ability of ProLncS to predict prognosis of HCC was compared with the previously published HCC prognosis model in the TCGA-LIHC (A), GSE76427 (B), GSE116174 (C), and GSE144269 (D). *P <0.05, **P <0.01, ****P <0.0001.


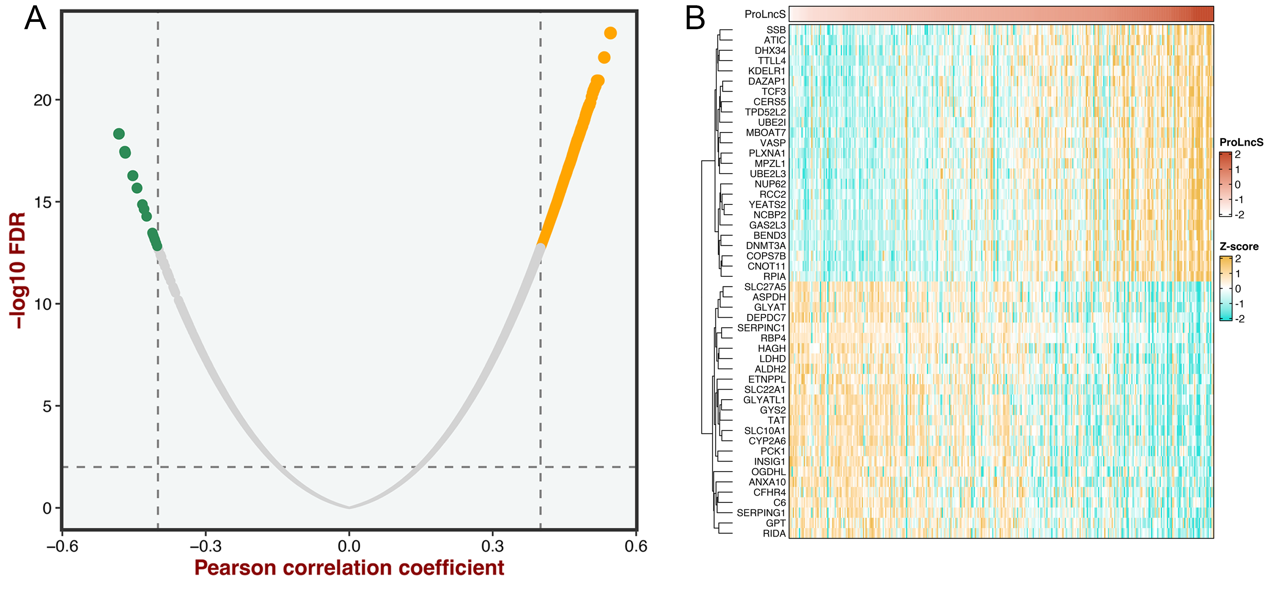


**Figure S8.** Pearson correlations between ProLncS and all genes. Distribution of 50 genes with ProLncS (genes with the top 25 positively and negatively correlated with ProLncS).


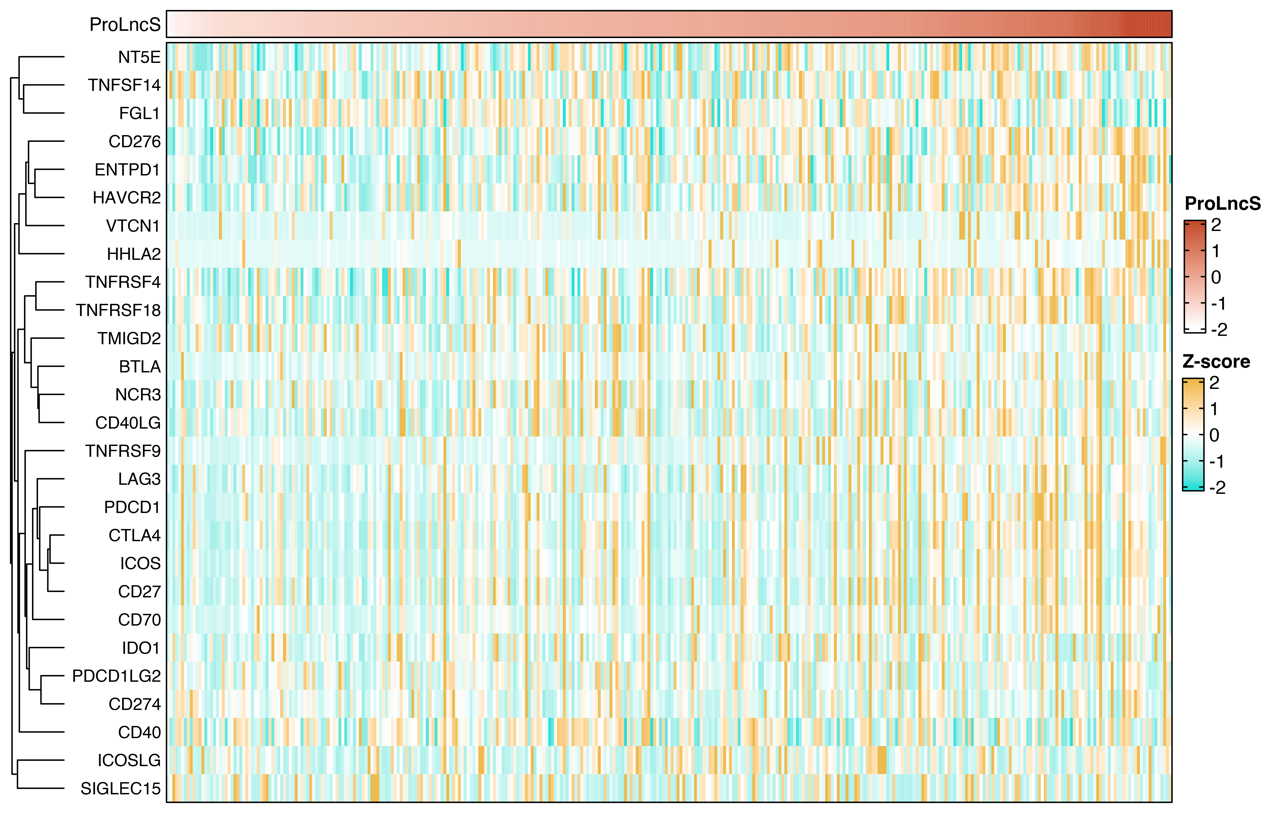


**Figure S9**. Distribution of 27 immune checkpoint molecules with ProLncS.
